# A Polarized Cell Model for Chikungunya Virus Infection: Entry and Egress of Virus Occurs at the Apical Domain of Polarized Cells

**DOI:** 10.1371/journal.pntd.0002661

**Published:** 2014-02-20

**Authors:** Pei Jin Lim, Justin Jang Hann Chu

**Affiliations:** Laboratory of Molecular RNA Virology and Antiviral Strategies, Department of Microbiology, Yong Loo Lin School of Medicine, National University Health System, National University of Singapore, Singapore, Singapore; Florida Gulf Coast University, United States of America

## Abstract

Chikungunya virus (CHIKV) has resulted in several outbreaks in the past six decades. The clinical symptoms of Chikungunya infection include fever, skin rash, arthralgia, and an increasing incidence of encephalitis. The re-emergence of CHIKV with more severe pathogenesis highlights its potential threat on our human health. In this study, polarized HBMEC, polarized Vero C1008 and non-polarized Vero cells grown on cell culture inserts were infected with CHIKV apically or basolaterally. Plaque assays, viral binding assays and immunofluorescence assays demonstrated apical entry and release of CHIKV in polarized HBMEC and Vero C1008. Drug treatment studies were performed to elucidate both host cell and viral factors involved in the sorting and release of CHIKV at the apical domain of polarized cells. Disruption of host cell myosin II, microtubule and microfilament networks did not disrupt the polarized release of CHIKV. However, treatment with tunicamycin resulted in a bi-directional release of CHIKV, suggesting that N-glycans of CHIKV envelope glycoproteins could serve as apical sorting signals.

## Author Summary

Polarized cells are found in many parts of the human body and are characterized by the presence of two distinct plasma membrane domains: the apical domain facing the lumen and the basolateral domain facing the underlying tissues. Polarized epithelial cells line the major cavities of our body, while polarized endothelial cells line the blood-tissue interface, both of which protect our body against the invasion of biological pathogens. Thus, many pathogens have to invade the monolayer of epithelial or endothelial cells in order to establish infection. During infection with Chikungunya virus, a mosquito vector bites a human host and inoculates the virus into the host's bloodstream. In recent epidemics of Chikungunya infection, more severe clinical manifestations such as neurological complications were observed. As such, we studied the infection of Chikungunya virus in polarized cells in an aim to provide explanations for the more severe pathogenesis observed.

## Introduction

Chikungunya virus (CHIKV) belongs to the *Alphavirus* genus of the *Togaviridae* family. It is a spherical, enveloped virus of 60 to 70 nm diameter that consists of the major structural proteins Capsid, E2 and E1, and a single-stranded, positive-sense RNA genome of 11.8 kb [1]. CHIKV was first isolated in Tanzania in 1952 during the earliest recorded Chikungunya epidemic [2] and has since caused outbreaks in East Africa, South Africa and Southeast Asia [3]. CHIKV re-emerged in the recent epidemic outbreaks, including the largest documented outbreak of CHIKV in the Indian Ocean islands of Mayotte, Mauritius, La Réunion, and the Seychelles between 2005 and 2006 [3] and in India between 2006 and 2008 [4]–[6]. Since then, CHIKV has caused outbreaks in many parts of the world, including Singapore [7], Malaysia [8] and Europe [9], [10].

CHIKV infection causes a range of clinical manifestations including high fever, headache, erythematous skin rash and incapacitating arthralgia [2]. Chikungunya disease is generally a self-limiting illness. However, the symptoms of the illness, rheumatological manifestations in particular, may be chronic and persist for several months. Additionally, the recent outbreaks of Chikungunya are associated with unusual severity and neurological complications such as encephalitis [1], [11]–[13].

Upon being bitten by a CHIKV-infected mosquito, CHIKV enters the bloodstream of the human host. It is currently unknown how CHIKV infection leads to encephalitis in the recent re-emergences of Chikungunya disease. One postulation is that CHIKV may migrate across the blood-brain barrier from the blood capillaries into the brain cells in order to cause neurological complications. The key structural elements of the blood-brain barrier are the tight junctions between adjacent brain capillary endothelial cells, which act as a barrier to prevent the diffusion and invasion of blood-borne pathogens from the bloodstream into the brain tissues and protect the brain from blood-borne toxic compounds and pathogens [14], [15].

Polarized cells, including the endothelial cells lining the brain capillaries, are characterized by the presence of two distinct plasma membrane domains: the apical domain facing the lumen and the basolateral domain facing the underlying tissues. Sorting machineries within polarized cells recognise apical and basolateral sorting signals such as peptide motifs and post-translational modifications on proteins and transport them specifically to their respective domains. Following polarized sorting of proteins, the tight junctions at cell-cell contacts prevent the movement of proteins between the two domains and maintain the unique protein composition of each domain [16]. These discrete membrane domains function in the selective absorption and release of many proteins and pathogens.

Polarized epithelial cells line the major cavities of the body and polarized endothelial cells line the blood-tissue interface, both of which form a selective barrier against the invasion of many pathogens. In order to establish infection, many pathogens have to invade the monolayer of epithelial or endothelial cells [17]–[20]. Several viruses have been shown to display polarized entry and/or release in cellular models. For example, the entry and release of West Nile Virus [18], Hepatitis A Virus [21] and Simian Virus 40 [17] occur preferentially at the apical surface. In comparison, the entry and release of Semliki Forest Virus [22], [23] and Vesicular Stomatitis Virus [19] occur preferentially at the basolateral surface.

The polarized infection of CHIKV may provide insights to the pathogenesis of the virus and the mechanisms involved in how the virus crosses the polarized blood-brain barrier in the establishment of neurological complications. Thus, this study aims to establish a polarized cellular model for CHIKV infection in order to investigate whether the entry and release of CHIKV is polarized. We also examined host cell and viral factors that may be involved in the polarized sorting and release of CHIKV at specific domains of the host cell.

## Methods

### Cell and virus cultures

Non-polarized African Green Monkey kidney epithelial cells (Vero) and polarized Vero C1008, both from American Type Culture Collection, were maintained in Dulbecco's modified Eagle's medium (DMEM) supplemented with 10% fetal calf serum (FCS). Human Brain Microvascular Endothelial Cells (HBMEC) from ScienCell were maintained in Endothelial Cell Medium (ECM) supplemented with 5% fetal bovine serum (FBS) and 1% endothelial cell growth supplement. The CHIKV strain used in this study, D1225Y08, was a kind gift from the Environmental Health Institute, National Environment Agency. D122508Y08 was isolated from the serum of a febrile patient during the 2007 to 2008 Chikungunya outbreaks in Singapore. The CHIKV122508 virus used in this study is a low passage virus that was cultured more than 5 passages in C6/36 cells derived from Aedes albopictus in Leibovitz's L15 medium supplemented with 2% FCS.

### Growth kinetics of CHIKV on HBMEC cells and Vero C1008 cells

4×10^4^ HBMEC cells (passage 2) were seeded on glass cover slips coated with 4 µg/cm^2^ fibronectin in 24-well plates. 5×10^4^ Vero C1008 cells were seeded on uncoated glass cover slips in 24-well plates. The cells were infected with CHIKV at a multiplicity of infection (MOI) of 10. CHIKV-infected HBMEC cells were maintained in ECM medium supplemented with 5% FBS and the supernatant was harvested at 0, 12, 24, 36, 48, 72, 96 and 120 hours post-infection (h.p.i.). CHIKV-infected Vero C1008 cells were maintained in DMEM medium supplemented with 2% FCS and the supernatant was harvested at 6-hours intervals up to 48 h.p.i.. The supernatants were subjected to viral plaque assays to quantify the virus titer. CHIKV-infected HBMEC and Vero C1008 cells were viewed under the differential interference contrast microscope at 24-hours and 12-hours intervals, respectively, to observe for morphology changes post-infection. Immunofluorescence assay was performed to detect CHIKV protein expression in the CHIKV-infected HBMEC and Vero C1008 cells.

### Assessment of cell monolayer integrity

The integrity of HBMEC, Vero C1008 and Vero cell monolayers was assessed to ensure that the cell monolayers remained intact and to prevent exchange of materials between the apical and basolateral chambers such that a polarized infection of CHIKV on the cells can be set up and that the virus titers obtained from the apical and basolateral chambers would be representative of the viruses released from the apical and basolateral domain, respectively.The integrity of HBMEC, Vero C1008 and Vero cell monolayers was assessed by measuring the trans-epithelial electrical resistance (TEER) using the Millicell-ERS apparatus (Millipore). Vero and Vero C1008 cell monolayers with TEER values between 30 and 70 Ω/cm^2^ and HBMEC cell monolayers with TEER values of approximately 20 Ω/cm^2^ were used for polarized infection studies. The integrity of the cell monolayers was assessed again post-infection by measuring the TEER detecting for ZO-1 tight junction proteins by immunofluorescence assay, and assaying for the permeability of the cell monolayers to FITC-dextran. In brief, FITC-dextran was applied to the apical chamber and incubated for 20 minutes, after which the percentage of FITC-dextran flow-through was calculated by the fluorescence reading in the basolateral chamber over the fluorescence reading in the apical chamber. 100 ng/ml tumour necrosis factor (TNF) was applied to the cells to increase the cel monolayer permeability as a positive control.

### Polarized infection of CHIKV

2×10^5^ HBMEC (passage 4), 5×10^4^ Vero C1008 and 5×10^4^ Vero cells were individually seeded on cell culture inserts with pores of 0.4 µm diameter (Greiner) and infected with CHIKV either apically or basolaterally at an MOI of 10. At 24 h.p.i., supernatants from the apical and basolateral chambers were collected separately for plaque assays to quantify the virus yields. To investigate the polarized entry of CHIKV, the total virus yield post-apical infection was compared to the total virus yield post-basolateral infection. The virus binding assay was also performed to further confirm the polarized entry of CHIKV. On the other hand, to investigate the polarized release of CHIKV, the amount of virus released into the apical and basolateral chambers were quantified separately by viral plaque assays and compared to determine whether the release of CHIKV in Vero C1008 and HBMEC cells is polarized. The cells were also fixed at 24 h.p.i., subjected to immunofluorescence assay and analyzed by confocal microscopy to visualize the localization of CHIKV protein expression.

### Virus binding assay

The virus binding assay was performed to analyze of the polarized entry of CHIKV in Vero C1008 cells. Vero and Vero C1008 cells were seeded on porous cell culture inserts and apically- or basolaterally-infected with CHIKV at an MOI of 10 at 4°C. After 1.5 hours incubation to allow CHIKV to bind to cell surface receptors, the cells were washed with 1×PBS four times and fixed with 4% paraformaldehyde. Immunofluorescence assay was performed to label CHIKV virus particles with FITC fluorochrome to quantify the amount of CHIKV bound to the cells. The specimens were viewed under the confocal microscope and analysed with the Imaris software to calculate the average number of FITC flurochrome spots per cell (DAPI-stained).

### Immunofluorescence assay

Cells were fixed with 4% paraformaldehyde for 10 minutes and permeabilised with 0.01% Triton X-100 for 15 minutes. The cells were then incubated with the desired primary antibodies at 37°C for 1 hour, followed by species-specific secondary antibodies at 37°C for 1 hour. In the growth kinetics studies, the samples were probed with primary antibodies against *Alphaviruses* (Santa Cruz sc-58088, 1∶250 dilution), followed by FITC-conjugated secondary antibodies (Millipore, 1∶500 dilution). In the polarized infection studies, the samples were co-labeled with primary antibodies against CHIKV E2 glycoproteins (1∶100 dilution) and FITC-conjugated secondary antibodies (Millipore, 1∶250 dilution) together with primary antibodies against ZO-1 proteins (Invitrogen, 1∶500 dilution) and Dylight-594-conjugated secondary antibodies (Thermo Scientific, 1∶100 dilution). In the virus binding assay, the samples were probed with primary antibodies against *Alphaviruses* (Santa Cruz, 1∶250 dilution), followed by FITC-conjugated secondary antibodies (Millipore, 1∶500 dilution). Cell nuclei were stained with 4′,6-diamidino-2-phenylindole (DAPI) at room temperature for 20 minutes. 1×PBS washes were performed after each incubation step. The samples were subsequently mounted onto glass slides using DABCO and viewed under the Olympus IX81 inverted microscope or Olympus FV1000 confocal microscope.

### qRT-PCR quantification of CHIKV viral RNA

Viral RNA was extracted (Qiamp viral RNA kit; Qiagen) from the virus supernatant collected from the upper and lower chambers of the cell culture insert after apical and basolateral infection with CHIKV. One-step SYBR green-based RT-PCR CHIKV viral RNA quantification was described previously [24] using the SYBR Green Quantitative RT-PCR Kit (Sigma-Aldrich, QR0100) in the ABI PRISM 7000 RT-PCR system. CHIKV samples were assayed with a concentration (250 nM) of the nsP2 primers (nsP2 forward primer: GGCAGTGGTCCCAGATAATTCAAG; nsP2 reverse primer: GTACATACCCCACCTAGATCTGTCG) in a 1× final concentration of SYBR green Taq Ready Mix for Quantitative RT-PCR (1× Taq DNA polymerase, 10 mM Tris-HCl, 50 mM KCl, 3.0 mM MgCl2, 0.2 mM dNTP, stabilizers) and 1× reference Dye. The RT-PCR conditions for the one-step SYBR green RT-PCR consist of a 20 minutes reverse transcription step at 44°C and then 2 minutes of Taq polymerase activation at 94°C, followed by 40 cycles of PCR at 94°C for 15 seconds (denaturation), 60°C for 1 minute (annealing and extension).

### Drug treatment assays

Cytochalasin B (Sigma) was used to reduce actin polymerisation rate by inhibiting the addition of actin monomers to the “barbed” end of microfilaments [25]. Nocodazole (Sigma) was used to inhibit the assembly of tubulin into microtubules [26]. Blebbistatin (Abcam) was used to inhibit myosin II by binding to myosin-ADP-P_i_ complex and intefering with phosphate release [27]. Tunicamycin (MP Biomedicals) was used to inhibit N-linked glycosylation of proteins [28].

The cell viability assay was first performed to assess the cytotoxicity of the various concentrations of cytochalasin B (0.1 to 4 µM), nocodazole (0.1 to 0.4 µM), blebbistatin (1 to 50 µM) and tunicamycin (0.2 to 4 µg/ml) by incubating Vero C1008 cells with the drugs in DMEM medium supplemented with 2% FCS for 24 hours. 0.1% DMSO was used as a non-treated control. After 24 hours incubation, one-tenth volume of alamarBlue reagent (Invitrogen) was added to the drug-treated cells and incubated for 2 hours at 37°C. Fluorescence measurements were performed at an excitation wavelength of 570 nm and an emission wavelength of 585 nm using a microplate reader (Infinite 200, Tecan). The percentage of cell viability was determined by comparing with the non-treated control.

Upon checking for cytotoxic effects, apically-infected Vero C1008 cells were fed with DMEM medium containing various concentrations of the drugs after the 1.5 hours virus adsorption period. Supernatants were collected after 24 hours for viral plaque assays to quantify the virus titer. The cells were analysed by immunofluorescence assay.

### Statistical analysis

Two-tailed student t-test was used to analyse the difference in total infectious virus titer post-apical infection and that post-basolateral infection, as well as the difference in infectious virus titer in the apical chamber and that in the basolateral supernatant.

## Results

### Growth kinetics of CHIKV in HBMEC and Vero C1008 cells

The susceptibility of Vero cells to CHIKV infection has been shown previously [29], [30]. However, it is not known if HBMEC and Vero C1008 cells are susceptible to CHIKV infection. As such, growth kinetics studies were performed to determine the susceptibility of HBMEC and Vero C1008 cells to CHIKV infection and to select appropriate time points to study the polarized infection of CHIKV.

**Figure 1:**
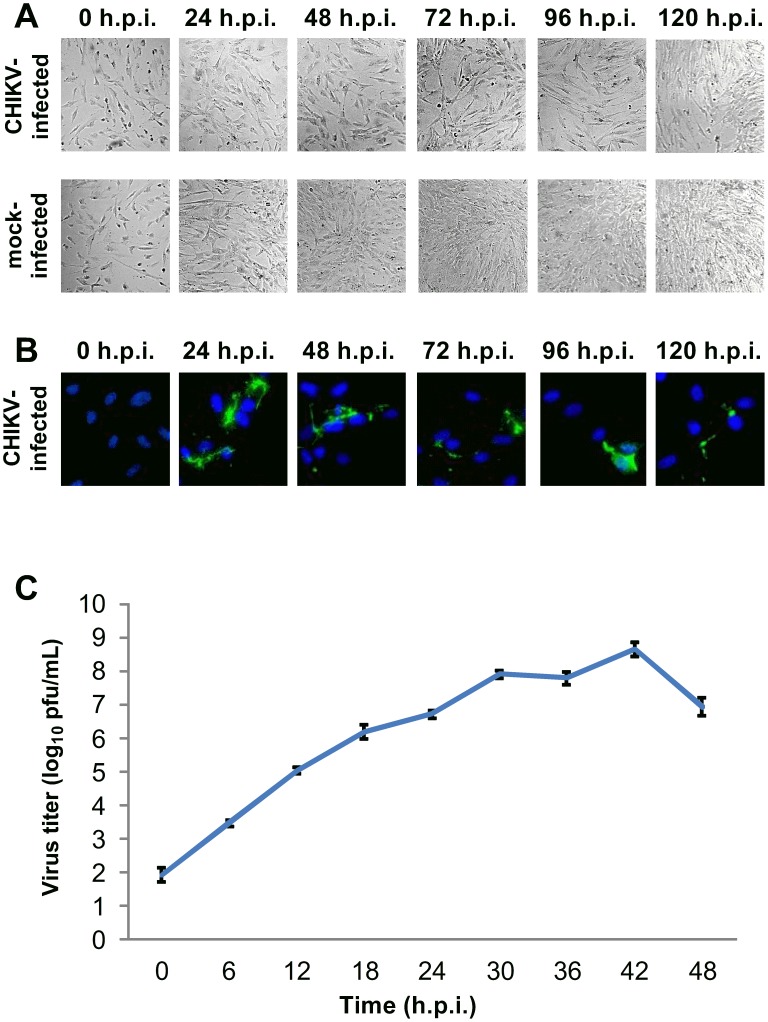
Growth kinetics of CHIKV in HBMEC at an MOI of 10. (**A**) CHIKV-infected HBMEC cells were viewed under DIC microscopy to observe for morphological changes. The cell density was lower in CHIKV-infected cells as compared to mock-infected cells from 48 h.p.i. onwards. (**B**) CHIKV-infected HBMEC cells were fixed at various intervals post-infection and immunofluorescence assay was performed to detect for CHIKV protein expression (green). Cell nuclei were stained with DAPI (blue). Moderate amounts of CHIKV protein expression were observed at 24 h.p.i. of HBMEC. (**C**) Quantification of infectious virus titer by plaque assay showed an increasing trend, with a peak in infectious virus titer at 36 h.p.i.. Vertical bars represent one standard deviation from the mean of three readings.

**Figure 2:**
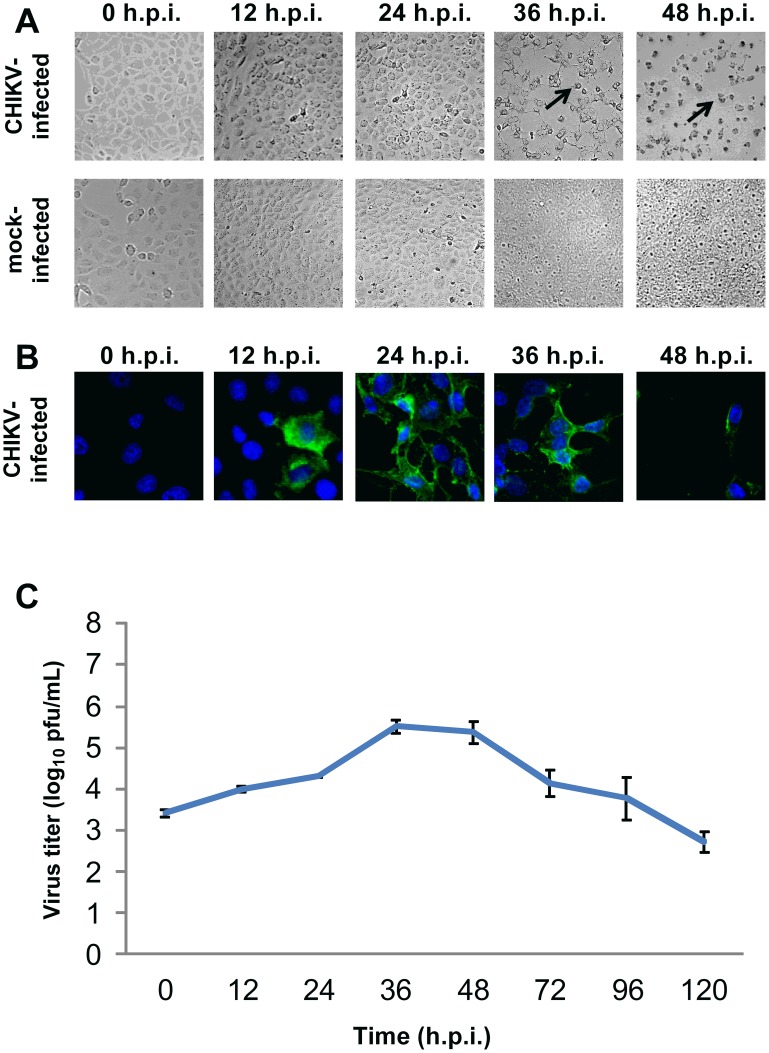
Growth kinetics of CHIKV in Vero C1008 at an MOI of 10. (**A**) CHIKV-infected Vero C1008 cells were viewed under DIC microscopy to observe for any morphological changes. Extensive CPE was observed from 36 h.p.i. onwards, as shown by the spindle-shaped and round appearance of cells (black arrows). (**B**) CHIKV-infected Vero C1008 cells were fixed at various intervals post-infection and immunofluorescence assay was performed to detect for CHIKV protein expression (green). Cell nuclei were stained with DAPI (blue). High amounts of CHIKV protein expression was observed at 24 and 36 h.p.i. of Vero C1008. (**C**) Quantification of infectious virus titer by plaque assay showed an increasing trend, with a peak in infectious virus titer at 42 h.p.i.. Vertical bars represent one standard deviation from the mean of three readings.

For CHIKV-infected HBMEC cells, the cell density upon CHIKV infection was lower than that of mock-infected cells (Figure 1A). Moderate amounts of CHIKV protein expression were detected at 24 h.p.i.. However, the amount of CHIKV antigen detected remained low up to 120 h.p.i. (Figure 1B). The infectious virus titer gradually increased with time post-infection, with a maximum titer of 3.4×10^5^ PFU/ml observed at 36 h.p.i. (Figure 1C). For CHIKV-infected Vero C1008 cells, extensive cytopathic effects (CPE) were observed from 36 h.p.i. onwards. The CHIKV-infected Vero C1008 cells were rounded and observed to be lifting off from the cell monolayer (Figure 2A). CHIKV antigen was detected as early as 12 h.p.i. and increased drastically at 24 and 36 h.p.i. (Figure 2B). The infectious virus titer also increased with time post-infection, with a maximum titer of 5.0×10^8^ PFU/ml observed at 42 h.p.i. (Figure 2C).

The growth kinetics studies confirmed that both HBMEC and Vero C1008 are susceptible to CHIKV infection, as shown by the increasing infectious virus titer with time and detection of CHIKV antigen by immunofluorescence assay. Additionally, we selected 24 h.p.i. as an appropriate time point for subsequent studies on the polarized infection of CHIKV in HBMEC and Vero C1008 cells for several reasons. Firstly, maximum amount of viral antigens were detected at 24 h.p.i. for HBMEC and at 24 and 36 h.p.i. for Vero C1008 cells via immunofluorescence assays. However, CPE was observed at 36 h.p.i. of Vero C1008, which will affect the integrity of the cell monolayer during the subsequent polarized infection studies. In comparison, the HBMEC and Vero C1008 cells remained well spread out in a monolayer at 24 h.p.i.. Thirdly, the infectious virus titers were observed to be increasing at 24 h.p.i.. From the above descriptions, 24 h.p.i. was selected as an appropriate time point for subsequent studies on the polarized infection of HBMEC and Vero C1008.

### Assessment of cell monolayer integrity

**Figure 3:**
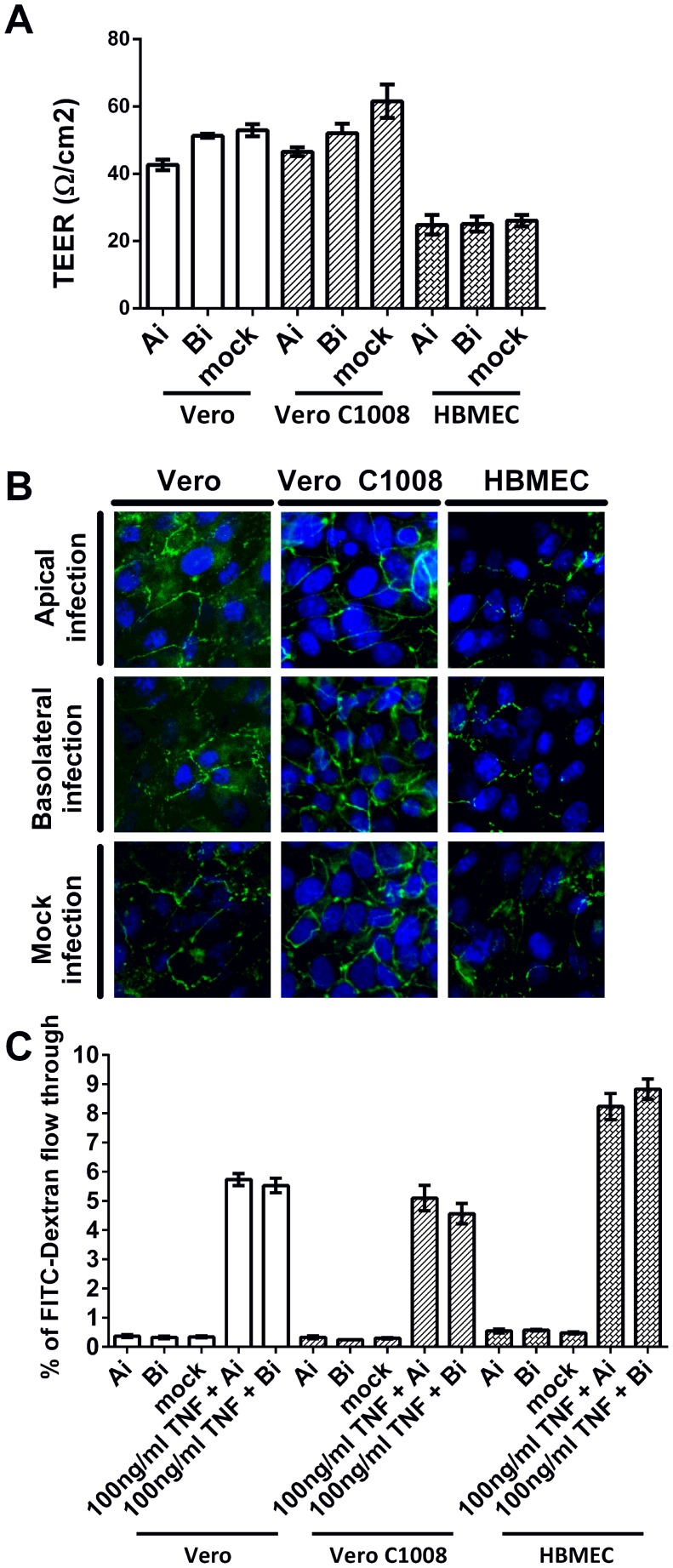
Cell monolayer integrity post CHIKV infection at an MOI of 10. (**A**) TEER measurements of Vero, Vero C1008 and HBMEC cell monolayers were taken at 24 h.p.i.. The TEER measurements post-apical and basolateral infection were comparable to that of mock-infected cells. Vertical bars represent one standard deviation from the mean of three readings. (**B**) Immunofluorescence assays demonstrated the expression of ZO-1 tight junction proteins (green) in apically-infected and basolaterally-infected Vero, Vero C1008 and HBMEC cells at 24 h.p.i.. The expression of ZO-1 proteins in infected cells is comparable to that of mock-infected cells. (C) The FITC-dextran permeability assays demonstrated that the integrity of Vero and Vero C1008 cell monolayers remained intact at 24 h.p.i., where the permeability of the cell monolayers to FITC-dextran remained low as compared to the TNF-treated cells.

Non-polarized Vero, polarized Vero C1008 and polarized HBMEC cells were seeded on cell culture inserts and infected with CHIKV either apically or basolaterally. The cell monolayers' integrity post-infection was determined by measuring the cell monolayers' trans-epithelial electrical resistance (TEER) and detecting ZO-1 protein expression via immunofluorescence assays. Upon apical and basolateral infection, the TEER of both Vero and Vero C1008 cell monolayers were within 40 and 70 Ω/cm^2^, which were comparable to that of mock-infected Vero and Vero C1008 cells (Figure 3A), demonstrating that the cell monolayers remained non-permeable post-infection to prevent exchange of materials between the apical and basolateral chambers. Thus, the infectious virus titer of the supernatant harvested from the apical chamber represents the amount of CHIKV released from the cells' apical domain, while the infectious virus titer of the supernatant harvested from the basolateral chamber represents the amount of CHIKV released from the cells' basolateral domain. Although the TEER of HBMEC cell monolayers were only between 20 and 30 Ω/cm^2^ post-apical and basolateral infection (Figure 3A), the TEER were comparable to that of mock-infected HBMEC cells. ZO-1 tight junction proteins were expressed in Vero, Vero C1008 and HBMEC cells at 24 h.p.i., and the expression of ZO-1 in infected cells was comparable to that in mock-infected cells (Figure 3B). The permeability of the Vero and Vero C1008 cell monolayers were also assayed by measuring the amount of FITC-dextran flow-through from the apical to the basolateral supernatant. The percentage of FITC-dextran flow-through was low for apically, basolaterally and mock-infected Vero and Vero C1008 cells as compared to the positive control cells treated with TNF (Figure 3C), further demonstrating that the integrity of the cell monolayers remained intact post-infection.

### Polarized infection of CHIKV

**Figure 4:**
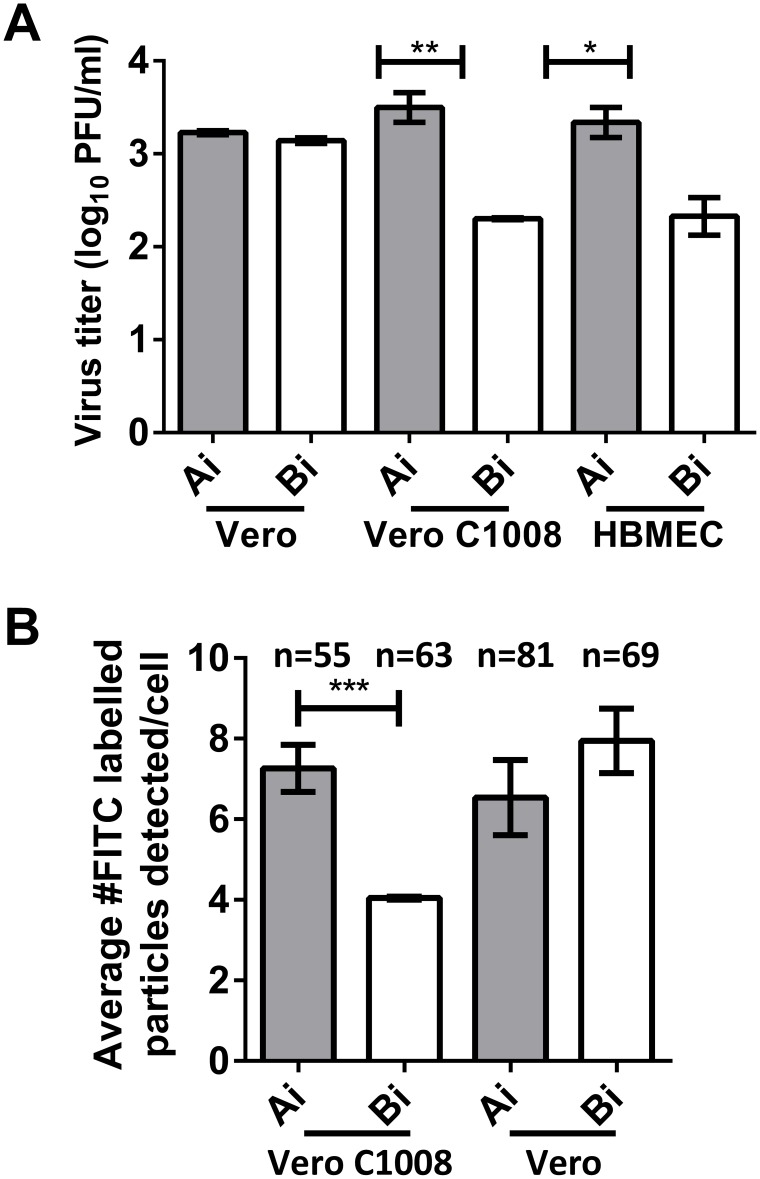
Polarized entry of CHIKV at apical plasma membrane domain. (**A**) The total virus yield at 24 h.p.i. of non-polarized Vero, polarized Vero C1008 and polarized HBMEC cells at an MOI of 10 was quantified by viral plaque assay. Entry of CHIKV is bi-directional in non-polarized Vero cells but occurs preferentially at the apical domain of polarized Vero C1008 and HBMEC cells. Two-tailed Student's *t*-test: * p<0.05, ** p<0.005. Vertical bars represent one standard deviation from the mean of three readings. (**B**) Virus binding assays show higher amount of CHIKV binding upon apical infection as compared to upon basolateral infection of polarized Vero C1008 cells. In contrast, the amount of CHIKV binding upon apical and basolateral infection of non-polarized Vero cells is similar. Two-tailed Student's *t*-test: *** p<0.001.

To investigate whether CHIKV enters Vero C1008 and HBMEC in a polarized manner, the total infectious virus titer at 24 hours post-apical infection was quantified by plaque assays and compared to the total infectious virus titer post-basolateral infection. Infection of non-polarized Vero cells was performed as a control. As expected, the total virus titer was similar between post-apical and post-basolateral infection of Vero cells. In contrast, the total virus titer post-apical infection of polarized HBMEC cells was 1.0 log units higher than that post-basolateral infection. Similarly, apical infection of Vero C1008 also resulted in a total virus yield of 1.2 log units higher than basolateral infection (Figure 4A). These data suggest that entry of CHIKV into Vero C1008 and HBMEC cells is polarized towards the apical domain. Furthermore, virus binding assays showed that the amount of CHIKV binding was higher upon apical inoculation of CHIKV as compared to basolateral inoculation of CHIKV in polarized Vero C1008 cells (Figure 4B). In contrast, the amount of CHIKV particles binding to non-polarized Vero cells were similar upon apical and basolateral inoculations. Therefore, these data further confirm the preferential entry of CHIKV at the apical domain of polarized Vero C1008 cells.

**Figure 5:**
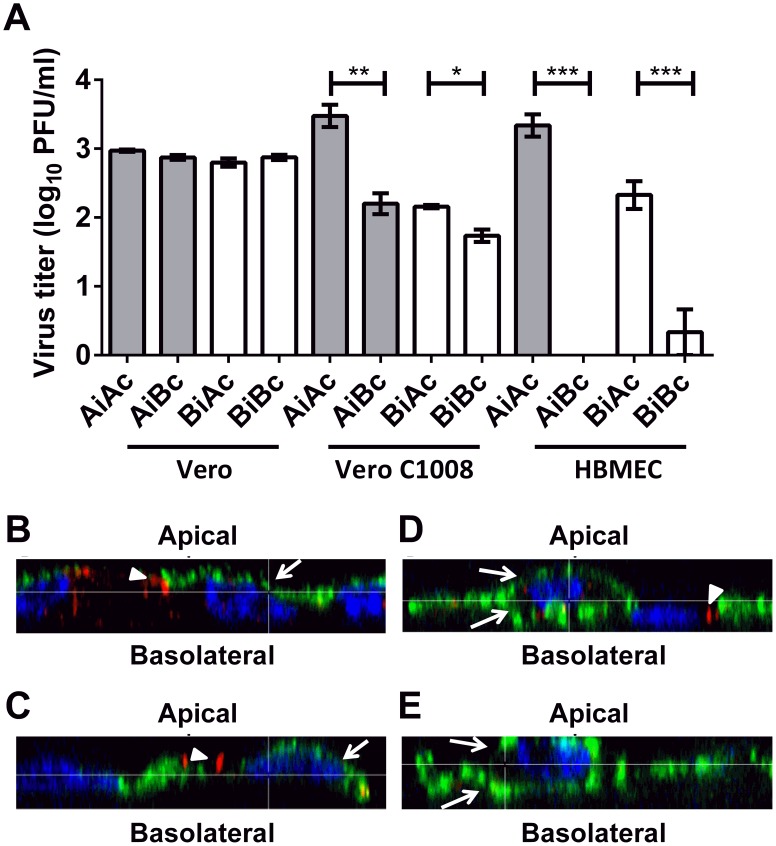
Polarized release of CHIKV at apical plasma membrane domain. (**A**) Infectious virus titer of supernatants collected from the apical and basolateral chambers at 24 h.p.i. of non-polarized Vero, polarized Vero C1008 and polarized HBMEC cells at an MOI of 10 were quantified by viral plaque assays. Release of CHIKV is bi-directional in non-polarized Vero cells but occurs preferentially at the apical domain of polarized Vero C1008 and HBMEC cells. Two-tailed Student's *t*-test: * p<0.05, ** p<0.005, *** p<0.001. Vertical bars represent one standard deviation from the mean of three readings. (**B**) Apically-infected Vero C1008, (**C**) basolaterally-infected Vero C1008, (**D**) apically-infected Vero and (**E**) basolaterally-infected Vero cells were co-labeled with antibodies against CHIKV E2 glycoprotein (green, arrows) and ZO-1 (red, arrowheads). Cell nuclei were stained with DAPI (blue). Z-stacked images show the polarized release of CHIKV towards the apical plasma membrane of Vero C1008 upon apical and basolateral infection. Release of CHIKV is bidirectional in non-polarized Vero cells.

Next, to investigate whether the release of CHIKV from Vero C1008 and HBMEC is in a polarized manner, infectious virus titer released into the apical chamber (AiAc) was compared to the infectious virus titer released into the basolateral chamber (AiBc) post-apical infection, while the infectious virus titer in the apical chamber (BiAc) was compared to the infectious virus titer in the basolateral chamber (BiBc) post-basolateral infection. When non-polarized Vero cells were apically- or basolaterally-infected with CHIKV, the infectious virus titer in the apical and basolateral chambers were similar (Figure 5A). In contrast, at 24 hours post-apical and basolateral infection of polarized Vero C1008 cells, the infectious virus titer was 1.3 and 0.3 log units higher in the apical chamber than in the basolateral chamber, respectively. Similarly, at 24 hours post-apical and basolateral infection of polarized HBMEC cells, the infectious virus titer was 3.3 and 1.5 log units higher in the apical chamber than in the basolateral chamber, respectively (Figure 5A). These data suggest the polarized release of CHIKV towards the apical domain of Vero C1008 and HBMEC cells.

To further illustrate the polarized release of CHIKV, immunofluorescence assays were performed on cells post-polarized infection with CHIKV using antibodies against the CHIKV E2 viral protein to visually inspect the membrane domain at which CHIKV is released from. Immunofluorescence assays were only performed on non-polarized Vero and polarized Vero C1008 cells as the growth kinetics studies showed that high CHIKV viral antigen expression could be detected in CHIKV-infected Vero C1008 cells (Figure 2B) but only low amounts of CHIKV antigens could be detected in CHIKV-infected HBMEC cells (Figure 1B). CHIKV-infected Vero and Vero C1008 cells were co-labeled with antibodies against CHIKV E2 viral protein (arrows, Figure 5B–E) and ZO-1 proteins (arrowheads, Figure 5B–E). ZO-1 proteins are markers for tight junctions between adjacent cells, as well as an apical marker to discriminate between the apical and basolateral domains [31]. The Z-section images demonstrated that upon both apical infection (Figure 5B) and basolateral infection (Figure 5C) of polarized Vero C1008 cells, CHIKV was preferentially released from the apical domain, where the CHIKV particles localized on the same membrane domain as ZO-1. In comparison, upon apical infection (Figure 5D) and basolateral infection (Figure 5E) of non-polarized Vero cells, CHIKV was released bi-directionally from both the apical and basolateral domains of Vero cells. Thus, the results further demonstrated the polarized release of CHIKV from the apical domain of polarized Vero C1008 cells. Of note, the ZO-1 staining depicted in red in Figure 5D appears to be on the basolateral side of the Vero cells and not on the apical side as would be expected. This is because Vero cells are non-polarised, hence the ZO-1 proteins could be located on either sides of the cell. In addition, the shape of the cell might just be that they tapered off at the sides, making the ZO-1 staining seem like it is at the basolateral side.

### Host cell factors facilitating apical sorting of CHIKV

The host cell cytoskeleton network and motor proteins have been shown to be play important roles in the polarized sorting of host cellular proteins and viral proteins [32], [33]. As such, we aimed to determine the involvement of host cell transport machineries in the polarized sorting of CHIKV towards the apical membrane for release. Cytochalasin B and nocodazole were used to inhibit actin polymerisation into microfilaments and tubulin polymerisation into microtubules, respectively. CHIKV-infected Vero C1008 cells were also treated with blebbistatin, a small molecule inhibitor of myosin II, to determine whether myosin II is involved in the apical sorting of CHIKV.

The alamarBlue assay was first performed to ensure that the drug concentrations used do not cause cytotoxic effects in Vero C1008. Results showed that the drug concentrations for cytochalasin B, nocodazole and blebbistatin did not result in cytotoxic effects (Figure S1). However, cell rounding was observed when 0.4 µM of nocodazole was added, which will disrupt the cell monolayer's integrity, allowing the exchange of materials between the apical and basolateral chambers. Hence, 0.1 to 0.3 µM nocodazole was used for the subsequent post-treatment studies.

**Figure 6:**
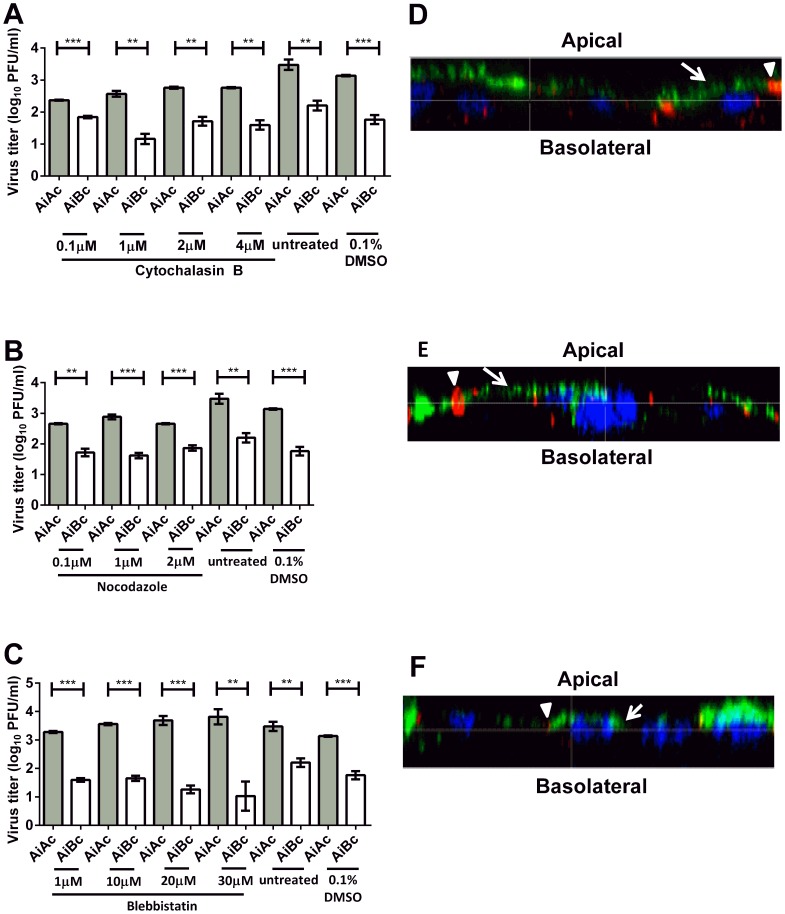
Release of CHIKV upon drug treatment to elucidate host cell factors involved in apical sorting of CHIKV. The infectious virus titer remained higher in the apical chamber than in the basolateral chamber upon treatment with (**A**) cytochalasin B, (**B**) nocodazole and (**C**) blebbistatin. One-tailed Student's *t*-test: ** p<0.005, *** p<0.001. Vertical bars represent one standard deviation from the mean of three readings. Apically-infected Vero C1008 cells were treated with (**D**) cytochalasin B, (**E**) nocodazole and (**F**) blebbistatin and co-labeled with antibodies against CHIKV E2 glycoprotein (green, arrows) and ZO-1 tight junction proteins apical markers (red, arrowheads). Cell nuclei were stained with DAPI (blue). Z-stacked images show the polarized release of CHIKV towards the apical plasma membrane of Vero C1008 cells upon treatment with cytochalasin B, nocodazole and blebbistatin.

Upon treating apically-infected Vero C1008 cells with cytochalasin B (Figure 6A), nocodazole (Figure 6B) and blebbistatin (Figure 6C), the infectious virus titers remained higher in the apical chamber than in the basolateral chamber. Additionally, confocal microscopy demonstrated that CHIKV was preferentially released from the apical domain of polarized Vero C1008 cells upon inhibition of actin polymerisation into microfilaments (Figure 6D), tubulin polymerisation into microtubules (Figure 6E), and myosin II (Figure 6F). These data suggest microfilaments, microtubules and myosin II are unlikely to be involved in the apical sorting of CHIKV in Vero C1008 cells, or may serve redundant functions in the apical sorting of CHIKV.

### CHIKV viral factors facilitating apical sorting of CHIKV

Besides the host cell factors, several apical sorting signals on apically-sorted proteins have been described to date, including glycosylphosphatidylinositol (GPI) membrane anchors, N-linked glycoproteins (N-glycans), NPXY motifs and YXXØ motifs [34]–[37]. Notably, the CHIKV envelope glycoproteins are N-glycosylated on asparagine residues N12 of E3 protein, N141 of E1 protein, and N263 of E2 protein [38]. Hence, to investigate viral factors involved in the sorting of CHIKV towards the apical domain of the host cells, CHIKV-infected Vero C1008 cells were treated with tunicamycin to inhibit N-glycosylation of the CHIKV envelope glycoproteins. The alamarBlue assay demonstrated that the concentrations for tunicamycin did not result in cytotoxic effects (Figure S1).

**Figure 7:**
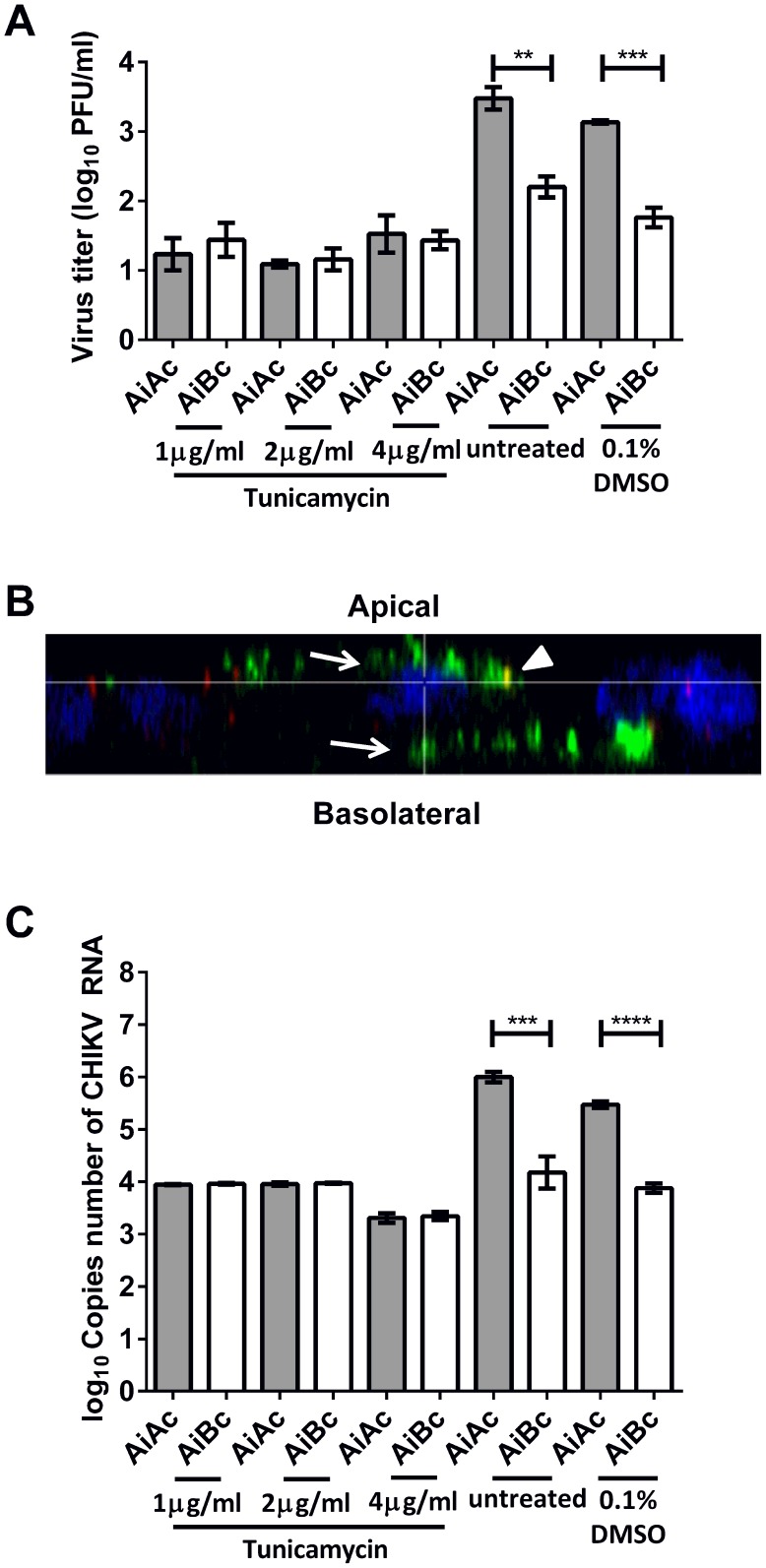
Release of CHIKV upon drug treatment to elucidate viral factors involved in apical sorting of CHIKV. (**A**) The infectious virus titers were similar in the apical and basolateral chambers upon treatment with tunicamycin. Vertical bars represent one standard deviation from the mean of three readings. (**B**) Apically-infected Vero C1008 cells were treated with tunicamycin and co-labeled with antibodies against CHIKV E2 glycoprotein (green, arrows) and ZO-1 tight junction proteins apical markers (red, arrowheads). Cell nuclei were stained with DAPI (blue). Z-stacked images show the bidirectional release of CHIKV upon treatment with tunicamycin. (C) The copies number of CHIKV RNA was higher in the apical chamber than in the basolateral chamber upon apical infection with CHIKV. Upon tunicamycin treatment, the copies number of CHIKV RNA was similar in the apical and basolateral chambers.

Interestingly, upon apical infection and tunicamycin treatment, similar infectious virus titers were obtained from the apical and basolateral chambers (Figure 7A). Additionally, confocal microscopy demonstrated that CHIKV was released from both apical and basolateral domains of polarized Vero C1008 cells in a bi-directional manner upon inhibition of N-glycosylation with tunicamycin (Figure 7B). qPCR assays were also performed to quantify the CHIKV RNA in the apical and basolateral chambers upon tunicamycin treatment as an alternative read-out for the amount of CHIKV particle released into the apical and basolateral chambers. Indeed, the qPCR results also demonstrated a non-polarized release of CHIKV upon tunicamycin treatment, where similar amounts of CHIKV RNA were detected in supernatants from both chambers (Figure 7C). These data suggest that the N-glycans of CHIKV glycoproteins may be involved in the polarized apical sorting of CHIKV.

## Discussion

### Vero C1008 cells as an *in vitro* model for the polarized infection of CHIKV

Several polarized cell lines have been used in the study of the polarized infection of viruses, including polarized African Green Monkey kidney epithelial cells (Vero C1008), polarized human intestinal epithelial cells (Caco-2) and polarized Madin-Darby canine kidney epithelial cells (MDCK) [17]–[20], [39], [40]. However, there have yet been any studies on the polarized infection of CHIKV. In this study, the polarized infection of CHIKV was examined in HBMEC and Vero C1008 cells. In both HBMEC and Vero C1008 cells, the entry and release of CHIKV was polarized towards the apical domain of the cells. However, the growth kinetics studies of CHIKV on HBMEC and Vero C1008 (Figures 1–2) demonstrated that Vero C1008 is more permissive to CHIKV infection than HBMEC, as shown by the higher increase in infectious virus titer post-infection and higher amount of CHIKV protein expression. In agreement with our results, Sourisseau and coworkers also reported a low infectivity of human brain microvascular endothelial cell line hCMEC/D3, whereby only 1% of the cell population were infected with CHIKV [29]. As such, we established Vero C1008 as a suitable and more convenient *in vitro* cell model for subsequent studies on the polarized infection of CHIKV and elucidation of mechanisms and sorting signals involved in the polarized sorting of CHIKV towards the apical domain.

### Polarized entry of release cellular pathogens

Epithelial and endothelial surfaces of the human body are a key component of the innate immune system because they act as a mechanical barrier against infection by pathogens. Polarized lung epithelial cells lining the respiratory tract are exposed to airborne pathogens. Polarized intestinal epithelial cells lining the small intestine are exposed to pathogens in the gastrointestinal tract. Polarized vascular endothelial cells lining the blood vessels are exposed to blood-borne pathogens. As such, these epithelial and endothelial surfaces are often the first point of contact between the human host and pathogens, and have to be breached by the pathogens in order to establish infection.

The polarized infection of viruses has been of research interest because it provides insights to the pathogenesis of the viruses. For example, infection with Simian Virus 40 (SV40) is characterized by the persistent infection of the rhesus monkey kidney, the presence of SV40 in the urine, as well as the absence of SV40 in the bloodstream [41]. This could be explained by the polarized entry and release of SV40 towards the apical domain of kidney tubular epithelial cells [17], thus restricting the infection of SV40 at the kidney. Additionally, the apical entry of Hepatitis A Virus (HAV) in polarized Caco-2 cells suggests that the intestinal epithelial cells can be infected by HAV present within the lumen of the gastrointestinal tract. Furthermore, the apical egress of HAV from Caco-2 cells may provide an explanation for the high shedding amount of HAV in the feces of HAV-infected patients. The apical egress of HAV from Caco-2 cells also suggests that epithelial cell infection is unlikely to result in penetration of HAV beyond the gastrointestinal epithelium. Thus, invasion of the liver by HAV may be dependent upon alternative mechanisms, such as transcytosis by specialized M cells in the distal ileum [21]. Furthermore, the entry of H1N1 and H5N1 Influenza viruses occurs bi-directionally in polarized alveolar epithelial cells, but releases predominantly at the apical domain facing the airways. The apical release of Influenza virus has been suggested to improve the transmissibility of Influenza virus by carrying the virus in respiratory droplets.

This study is the first to demonstrate the polarized entry and release of CHIKV towards the apical domain of polarized cells. The polarized entry of CHIKV at the apical domain of HBMEC and Vero C1008 cells suggests that the CHIKV receptors could be pre-dominantly sorted to the apical domain, allowing CHIKV to attach to the receptors and enter the polarized cells preferentially at the apical plasma membrane. Furthermore, the polarized release of CHIKV at the apical plasma membrane of HBMEC and Vero C1008 cells suggest that CHIKV structural proteins may contain apical sorting signals that direct their sorting towards the apical domain.

The polarized entry and release of CHIKV towards the apical plasma membrane of HBMEC may implicate that it is unlikely for CHIKV to gain access into the brain to cause neurological complications by apical entry from the blood into the brain microvascular endothelial cells and basolateral egress from the endothelial cells into the brain tissues. As such, other mechanisms could be explored to understand how CHIKV enters the central nervous system. For example, the entry and release of West Nile Virus (WNV) occurs at the apical domain of polarized cells, which is similar to CHIKV infection, and infections with WNV have been associated with neurological complications too. WNV causes neurological complications via the release of tumor necrosis factors, which transiently increases the permeability of the blood-brain barrier, thereby allowing WNV to diffuse across the blood-brain barrier from the bloodstream into the brain [42]. In another example, Venezuelan equine encephalitis virus (VEEV), a member of the *Alphavirus* genus, is also associated with encephalitis. VEEV infects the olfactory sensory neurons and spreads by retrograde neuronal dissemination into the brain to initiate viral replication in the brain initially. Subsequently, VEEV induces the biphasic opening of the blood-brain barrier and allows a second wave of VEEV from the periphery to enter the brain [43]. In addition, Couderc and coworkers [44] demonstrated that primary choroid plexus epithelial cells were highly susceptible to CHIKV infection in a polarized manner similar with what is demonstrated in our findings, with preferential entry via the apical domain. Thus, an alternative mechanism by which CHIKV infects the brain is through the cerebrospinal fluid produced through the choroid plexuses.

Nevertheless, the results of the polarized studies of CHIKV highlight that CHIKV infection could attain high viremia by apical entry into vascular endothelial cells lining the blood vessels, multiplying within the endothelial cells to high quantities, and releasing the newly synthesized CHIKV progenies back into the bloodstream at the apical domain. The high viremia in the bloodstream may aid the transmission of CHIKV as the mosquito vectors bite the CHIKV-infected patients and transfer the viruses to other human hosts during their next blood meal.

### Host cell factors involved in the polarized sorting of CHIKV towards the apical domain

The cytoskeleton network and motor proteins in host cells have been shown to be involved in the polarized sorting of host cellular proteins and viral proteins [32], [33]. For example, the apical sorting of Measles Virus matrix protein is dependent on microfilaments [45] while apical sorting of West Nile Virus envelope proteins is dependent on microtubules [18]. In addition, myosin II motor proteins facilitate the apical sorting of Bile Salt Export Protein (BSEP), so that BSEP can perform its transporter function to secrete bile acids at the apical membrane domain [46]. However, this study demonstrated that individual inhibition of microfilament formation, microtubules formation and myosin II functions do not disrupt the apical sorting of CHIKV. This suggests that the microfilament and microtubule cytoskeleton networks and motor proteins may perform redundant roles in the apical sorting of CHIKV, such that the individual inhibition of one of the cytoskeleton or motor protein factors do not disrupt apical sorting of CHIKV. Furthermore, the apical sorting of CHIKV may involve other host cell mechanisms such as Rab proteins. For example, Rab4 proteins are involved in the redistribution of the transferrin receptor from the basolateral plasma membrane to the apical plasma membrane during the indirect sorting of transferrin receptor to the apical domain [47]. On the other hand, Rab 11 and Rab 14 proteins are involved in the apical sorting of the ribonucleoprotein and hemagglutinin of Influenza virus [48], [49]. Thus, the host cell factors involved in the apical sorting of CHIKV in polarized cells remain to be studied.

### Viral factors involved in the polarized sorting of CHIKV towards the apical domain

N-glycans have been widely described as sorting signals that direct the apical sorting of proteins [36], [50], such as the Bile Salt Export Protein [28]. Interestingly, inhibition of N-glycosylation by the addition of tunicamycin resulted in a bi-directional release of CHIKV from polarized Vero C1008 cells. These data suggest that N-glycans on the E1, E2 and E3 envelope glycoproteins of CHIKV could serve as apical sorting signals that direct the trafficking of CHIKV preferentially towards the apical domain. In comparison, previous studies have shown that release of Vesicular Stomatitis Virus (VSV) and Influenza virus remained polarized despite inhibition of glycosylation using tunicamycin [51]. Thus, the polarized sorting of each virus may depend on different apical sorting signals.

The mechanism(s) by which N-glycans mediate the apical sorting of glycoproteins is not well-understood currently. Nevertheless, vesicular integral-membrane protein 36 (VIP36), a type of mannose-binding lectin, has been proposed to be an apical sorting receptor that binds high-mannose-type N-glycans and facilitate apical sorting. VIP36 is predominantly located at the endoplasmic reticulum-Golgi intermediate compartment (ERGIC). The apical membrane content of VIP36 is twice as high as the basolateral content in MDCK cells, and the plasma membrane glycoproteins recognized by VIP36 are also twofold higher in the apical membrane compared to the basolateral membrane [52]. Thus, binding of VIP36 to high-mannose-type N-glycans may facilitate the sorting of glycoproteins carrying the high-mannose-type N-glycans at the endoplasmic reticulum into vesicles destined for the apical plasma membrane.

The drug treatment assays also showed that tunicamycin resulted in a reduction of total infectious CHIKV released. A similar reduction in VSV and Influenza infectious virus titer was also observed when the virus-infected cells were treated with tunicamycin [51], although the release of VSV and Influenza virus remained polarized towards the apical domain. This reduction in infectious virus titer could be due to the inhibition of N-glycosylation of the viral envelope glycoproteins, which might affect their ability to bind to the virus receptors on host cells during infection. Thus, tunicamycin might have inhibited the N-glycosylation of the envelope glycoproteins and interfered with the infectivity of the newly synthesized enveloped virus progenies, resulting in the production of lower infectious virus titer. As such, further studies is in progress to elucidate the role of N-glycans in the apical sorting of CHIKV in a polarized cell model.

## Supporting Information

Figure S1 **Assessment of cytotoxicity of drugs on Vero C1008.** AlamarBlue assay was performed to test for cell cytotoxicity of the range of concentrations of (**A**) cytochalasin B, (**B**) nocodazole, (**C**) blebbistatin and (**D**) tunicamycin used. The percentage of cell viability remains at approximately 100%, indicating that the drugs are not cytotoxic to Vero C1008 cells. Vertical bars represent one standard deviation from the mean of three readings.
(TIF)
